# A Novel Antiserum Against a Predicted Human Peripheral Choline Acetyltransferase (hpChAT) for Labeling Neuronal Structures in Human Colon

**DOI:** 10.3389/fnana.2019.00037

**Published:** 2019-04-16

**Authors:** Jean-Pierre Bellier, Pu-Qing Yuan, Kenichi Mukaisho, Ikuo Tooyama, Yvette Taché, Hiroshi Kimura

**Affiliations:** ^1^Molecular Neuroscience Research Center, Shiga University of Medical Science, Otsu, Japan; ^2^CURE/Digestive Diseases Research Center, Vatche and Tamar Manoukian Digestive Diseases Division, Department of Medicine, UCLA David Geffen School of Medicine, Los Angeles, CA, United States; ^3^VA Greater Los Angeles Health System, Los Angeles, CA, United States; ^4^Department of Pathology, Shiga University of Medical Science, Otsu, Japan

**Keywords:** colon, cholinergic neurons, human, peripheral choline acetyltransferase, pChAT

## Abstract

Choline acetyltransferase (ChAT), the enzyme synthesizing acetylcholine (ACh), has an exon-skipping splice variant which is expressed preferentially in the peripheral nervous system (PNS) and thus termed peripheral ChAT (pChAT). A rabbit antiserum previously produced against rat pChAT (rpChAT) has been used for immunohistochemistry (IHC) to study peripheral cholinergic structures in various animals. The present study was undertaken to develop a specific antiserum against a predicted human pChAT (hpChAT) protein. A novel mouse antiserum has been successfully raised against a unique 14-amino acid sequence of hpChAT protein. Our Western blot using this antiserum (termed here anti-hpChAT serum) on human colon extracts revealed only a single band of 47 kDa, matching the deduced size of hpChAT protein. By IHC, the antiserum gave intense staining in many neuronal cells and fibers of human colon but not brain, and such a pattern of staining seemed identical with that reported in colon of various animals using anti-rpChAT serum. In the antibody-absorption test, hpChAT-immunoreactive staining in human colon was completely blocked by using the antiserum pre-absorbed with the antigen peptide. Double immunofluorescence in human colon moreover indicated that structures stained with anti-hpChAT were also stained with anti-rpChAT, and vice versa. hpChAT antiserum allowed the identification of cell types, as Dogiel type cells in intramural plexuses, and fiber innervation of colon muscles and mucosae. The present results demonstrate the specificity and reliability of the hpChAT antiserum as a novel tool for immunohistochemical studies in human colon, opening venues to map cholinergic innervation in other human PNS tissues.

## Introduction

Acetylcholine (ACh) was the first molecule identified to act as a neurotransmitter (Loewi and Navratil, 1926). Now, almost a hundred years later, we still lack the methodology to visualize ACh itself in nervous tissue (Anglade and Larabi-Godinot, 2010). So far, the histochemical identification of ACh-synthesizing cells relies exclusively on immunohistochemistry (IHC) using antibodies against proteins specifically associated with ACh metabolism, namely choline acetyltransferase (ChAT; Kimura et al., 1980), vesicular ACh transporter (Ichikawa et al., 1997; Roghani et al., 1997; Schäfer et al., 1998), and high-affinity choline transporter (Misawa et al., 2001).

ChAT (E.C. 2.3.1.6.) is the enzyme responsible for ACh synthesis. ChAT-IHC has been most widely used as a reliable marker to visualize cell bodies and fibers in the central nervous system (CNS; Kimura et al., 1980; Eckenstein et al., 1981; Levey et al., 1981), while IHC for vesicular ACh transporter or high-affinity choline transporter preferentially labels cholinergic fibers and terminals but not cell bodies clearly (Ichikawa et al., 1997; Misawa et al., 2001). In addition, ChAT-IHC shows a high morphological resolution that has enabled the detailed mapping of cholinergic pathways in the CNS of many species including rat and human (Mesulam, 1990; Woolf, 1991). This contrast with the peripheral nervous system (PNS), where ChAT-IHC often gives vague and faint staining of cholinergic cells and fibers. A few antibodies have been used to label peripheral cholinergic structures in the rat (Sann et al., 1995), guinea-pig (Schemann et al., 1993) and human (Porter et al., 1996, 2002; Ratcliffe et al., 1998; Neunlist et al., 2003; Beck et al., 2009). However, in the human PNS, the detailed organization of cholinergic structures remains unclear as mentioned in studies reporting failures in staining of notable components such as cholinergic nerves in intestinal mucosae (Porter et al., 1996; Ratcliffe et al., 1998). This raises a question of whether ChAT protein in the PNS differs from that in the CNS.

**Figure 1:**
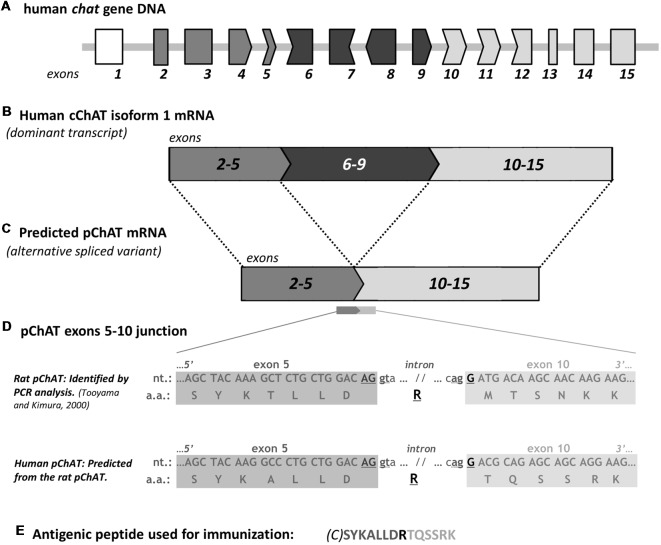
A schematic diagram for predicting human peripheral choline acetyltransferase (pChAT). The human *chat* gene contains 15 exons **(A)**. Some of these exons have nucleotides base residues; one or two bases at 3’-end of an exon and two or one base at 5’-end of the following exon. Across the alternative splice junction between two such exons, three bases joined to encode an amino acid. As to nucleotide residues, six types of exon are shown in **(A)** with respective shapes of their enclosing frames; type 1 (no residue; exons 2, 3 and 13–15), type 2 (two residues at 3’-end; exons 4 and 9), type 3 (one residue at 5’-end plus 2 residues at 3’-end; exons 5, 10 and 11), type 4 (one residue at 5’-end; exons 6 and 12), type 5 (one residue at 3’-end; exon 7), and type 6 (two residues at 5’-end; exon 8). By full-length alternative splicing of exons 2–14, common ChAT (cChAT) isoform 1 mRNA is formed **(B)**. In the rat, a similar framework has been found regarding both *chat* gene and cChAT mRNA splicing. We have therefore predicted the alternative splicing for human pChAT (hpChAT) mRNA, which is encoded in 10 exons **(C)**, produced by four-exons skipping (exons 6–9), in a similar way to that identified in rat (Tooyama and Kimura, 2000). The difference in nucleotide sequence at the spliced junction of exon 5 and exon 10 between rat and human is shown in **(D)**. In the rat, arginine (R) is encoded by three nucleotides across the splice junction; two (AG) from the 3’ end of exon 5 and one (G) from 5’ end of exon 10. In human, arginine (R) is incidentally also formed in exactly the same way. There are amino acids encoded similarly by two spliced exons, six in cChAT and four in predicted pChAT of both rat and human, as defined or predicted on the basis of two databases (“amino acids encoded across a splice junction” in NCBI CCDS database release 22, and “residue overlap splice site” in Ensembl database release 94). Note the two sets of two nucleotides, “gt” at the 5’ end of the intron after exon 5 and “ag” at the 3’ end of the intron before exon 10, which appear to serve as the splice donor and acceptor sites, respectively. Thanks to this splicing machinery, parts of the original cChAT reading frame are perfectly retained in pChAT. The peptide sequence used as an antigen for raising hpChAT is shown in **(E)**, where C in parenthesis is extra added cysteine for conjugation with a carrier protein. For simplification, other three upstream exons coding for the larger cChAT isoform 3 and vesicular acetylcholine (ACh) transporter are omitted from the human *chat* gene in the diagram presented. nt, nucleotides; a.a. amino acids.

Molecular genetic analysis has indicated that, in the rat, ChAT protein of about 72 kDa is encoded by the *chat* gene via alternatively spliced mRNA (Brice et al., 1989; Ishii et al., 1990). The transcript from this gene consists of 14 exons (exons 2–15, Figure 1). In 2000, on the other hand, Tooyama and Kimura identified a novel alternative splice variant mRNA from the *chat* gene in the rat PNS (Tooyama and Kimura, 2000). The variant lacks four exons (exons 6–9), likely arising from an exon-skipping mechanism of alternative splicing. This transcript encodes a variant ChAT protein with an expected molecular weight of 49 kDa (Figure 1). Because the amino acid composition of the variant ChAT protein is included in the largest known ChAT protein except for an arginine at a new splice junction of exons 5 and 10, the production of a specific antiserum against the variant turned to be technically challenging. This issue was solved when a rabbit antiserum was raised against a peptide (41 amino acids) encoded by the nucleotide sequence across the splice junction. The sequence is distinctive in the variant ChAT but absent in the known ChAT (Figure 1). IHC studies in rats using the antiserum showed the presence of the variant ChAT in neuronal cells and fibers of the PNS. The tissues and organs containing these immunoreactive structures include parasympathetic ganglia, heart, iris and enteric intramural ganglia, all of them are known to contain cholinergic cells or fibers (see the review by Bellier and Kimura, 2011). In contrast, no positive structures, with a few exceptions, were recognized in the CNS. The novel variant was thus named peripheral ChAT (pChAT). To avoid confusion, the known form of ChAT was called common ChAT (cChAT) because of its presence in both CNS and PNS (Tooyama and Kimura, 2000). This antiserum against rat pChAT (rpChAT) has been successfully used to study presumed cholinergic cells and fibers in the PNS not only in the rat but also in various species, including vertebrates (mouse, guinea-pig, sheep, pig and monkey) and invertebrates (molluscan octopus; Chiocchetti et al., 2003, 2004; Brehmer et al., 2004; Yuan et al., 2005; Wang et al., 2007; Koga et al., 2013; Sakaue et al., 2014; Bellier et al., 2017). The rpChAT antiserum has been further used for studying animal models of diseases by IHC (Tangsucharit et al., 2012; Wang et al., 2012; Duboc et al., 2016; Rice et al., 2017; Uranga et al., 2017).

The present study aims at developing an antiserum against the predicted human pChAT (hpChAT) to investigate presumed cholinergic structures in the human PNS. Although the existence of hpChAT has not been known, the available information on the human *chat* gene allows us to predict the sequence of hpChAT mRNA as a human counterpart of rpChAT. A tetradecapeptide selected from the predicted sequence is found in this study to be a successful antigen to raise a very high titer antiserum against the predicted hpChAT protein. Here, we report the details of hpChAT antibody characterization and its use to visualize neuronal structures positive for the antiserum in human colon. The colon was chosen because of important cholinergic innervation demonstrated by functional studies in a wide variety of animal species (see Furness, 2006) and also because of the dense cholinergic innervation shown by IHC using rpChAT antiserum in rats and mice (Nakajima et al., 2000; Tooyama and Kimura, 2000; Yuan et al., 2005; Wang et al., 2007). Many potential applications exist for this new antiserum, in particular, to study cholinergic structures in the PNS, including the enteric nervous system, of normal and diseased human tissues.

## Materials and Methods

### Production of Antisera Against Human pChAT

For immunization antigen, we have used a synthetic peptide CSYKALLDRTQSSRK (Japanese Patent 04412018JP) consisting of a 14-amino acid sequence which occurs in both wings extending before and after the junction of exons 5 and 10 in the predicted hpChAT protein, and the N-terminal extra added cysteine (Figure 1). This amino acid is to conjugate the peptide with the immunogenic carrier protein, keyhole limpet hemocyanin (KLH), using the Imject Maleimide-Activated mcKLH Spin kit (Thermo Fisher Scientific, Rockford, IL, USA) according to the manufacturer’s instruction.

This study was carried out in accordance with the recommendations of “NIH Guide for Care and Use of Laboratory Animal protocols” and of the Institutional Animal Care and Use Committee of Shiga University of Medical Science (SUMS). The protocol was approved by SUMS. Six adult male Balb/c mice (5-weeks-old) were purchased from Clea Japan Inc. (Konan, Japan). Each mouse (labeled H1-H6) received subcutaneous injections of 0.1 ml of TiterMax Gold adjuvant (Sigma-Aldrich, Saint-Louis, MO, USA) containing 5 mg/ml KLH-conjugated peptide into multiple sites of the neck and back skin with at a 2-week interval. Antisera, collected 4 days after immunization or booster injections were tested by IHC on sections of human colon. After 9–13 booster injections, the whole blood was collected from the heart under deep anesthesia with an intraperitoneal injection of sodium pentobarbital (80 mg/kg, Somnopentyl, Kyoritsu Seiyaku, Tokyo, Japan). After exsanguination, animals were euthanized by intracardiac injection with a lethal dose of sodium pentobarbital (150 mg/kg). The antiserum was isolated from collected mouse blood using a blood-serum separation device (Nipro, Osaka, Japan). All mice immunized gave high-titer antisera capable of labeling positive structures in human colon by IHC (Supplementary Figure S1). Among them, the mouse H3 gave the highest titer antiserum after 13 booster injections. Then, this H3 antiserum, termed here hpChAT antiserum, has been used in the present study.

On the other hand, it is noteworthy that we have also used three male rabbits (6-weeks-old; Clea Japan Inc.) to raise antisera against the same antigen as the above. For reasons yet unknown, however, no rabbits gave suitable antisera for IHC even after extended booster injections (data not shown).

### Human Tissues

This study was carried out in accordance with the recommendations of the SUMS Committees for Biosafety and Ethics and of the University of California at Los Angeles (UCLA) Institutional Review Board. The protocol was approved by the SUMS Committees for Biosafety and Ethics (Approval number 29-274) and the UCLA Institutional Review Board (IRB #17-001686). All subjects gave written informed consent in accordance with the Declaration of Helsinki.

Human small and large intestines (duodenum, ileum and ascending colon) from three subjects, and upper cervical ventral spinal cord and striatum from another two subjects, dissected at pathological autopsy in SUMS, were used for Western blot and histological analyses. All non-disease affected tissues were obtained between 3–7 h postmortem. Healthy margins of sigmoid colon (about 5 × 3 cm) with full-thickness, collected at UCLA from two patients with diverticulitis, were used for whole mount preparations.

### Western Blotting

Western blot analysis was performed as described previously with a slight modification (Bellier and Kimura, 2007). Human ascending colon and ventral spinal cord tissues were quickly frozen in liquid nitrogen and stored at −80°C until use. The frozen tissues were homogenized (10% w./v.) in 50 mM Tris–HCl (pH 7.4) containing 0.5% Triton-X100 and protease inhibitor cocktail (P-2714, Sigma-Aldrich) using a Polytron (Kinematica Gmbh, Kriens-Luzern, Switzerland). After centrifugation (12,000 *g* × 20 min) at 4°C the supernatant was collected, and the protein concentration was determined using a detergent-compatible protein assay kit based on the Bradford’s method (Bio-Rad Laboratories, Tokyo, Japan). An aliquot of the supernatant containing approximately 20 μg protein and a molecular weight marker (Protein Ladder One, Nacalai Tesque, Kyoto, Japan) were loaded on a 5%–20% precast PAGE-SDS gels (Nacalai Tesque). After electrophoresis, proteins were transferred to a nitrocellulose membrane (Hybond-ECL, GE Healthcare, Buckinghamshire, UK) for immunostaining with hpChAT antiserum developed in this study. In some cases, nitrocellulose membranes bearing blotted proteins were fixed for 30 min at 60°C with humid paraformaldehyde (PFA) vapor in a sealed container. Such PFA fixation has been shown to improve the retention rate of proteins blotted (Lee and Kamitani, 2011; Preterre et al., 2015), and also to modify a protein recognizable by antibodies through conformation change (Fowler et al., 2011). We used PFA fixation to enhance the intensity of positive staining on colon extracts and reduce the intensity of non-specific staining on spinal cord extracts. After extensive washing in 0.1 M Tris–HCl buffered saline (pH 7.4, TBS), the membranes were exposed for 1 h to 10% fat-free milk in TBS to block non-specific binding, followed by overnight incubation at room temperature with hpChAT antiserum (diluted 1:2,500), and for 1 h at room temperature with peroxidase-labeled goat anti-mouse IgG or peroxidase-labeled goat anti-rabbit IgG (each diluted 1:50,000; Jackson ImmunoResearch Laboratories, West Grove, PA, USA). TBS containing 0.5% Tween-20 was used to dilute all reagents and wash membranes after each step. The peroxidase activity was revealed by chemiluminescence using Chemi-Lumi One Super (Nacalai Tesque, Kyoto, Japan), then recorded using a LAS-3000 Luminoimager (Fujifilm, Tokyo, Japan).

### Tissue Preparation

Tissues of human duodenum, ileum, ascending colon and striatum were fixed by immersion for 4–7 days at 4°C in a solution containing 4% PFA and 0.1 M sodium phosphate buffer (PB, pH 7.4). The tissues were then placed in 0.1 M PB containing 15% sucrose for at least 24 h, frozen in Tissue-Tek optimum cutting temperature compound (Sakura, Tokyo, Japan), and cut into 28-μm-thick transverse or longitudinal sections in a cryostat. Sections were stored in 0.1 M PB containing 15% sucrose. Before staining, sections in a free-floating state were incubated in 0.01 M PB (pH 7.4) containing 0.9% NaCl and 0.3% Triton X-100 (PBST) for at least 24 h at 4°C to improve antibody permeability of tissues.

### Whole-Mount Preparations of Longitudinal Muscle/Myenteric Plexus

Human sigmoid colonic specimens were pinned flat and fixed overnight in 0.1 M PB containing 4% PFA. After washing in 0.01 M PBS, tissue samples were processed for whole-mount preparations of the longitudinal muscle/myenteric plexus and the submucosa containing submucosal plexus. Briefly, the mucosa was scraped off, the submucosa was collected, and the circular muscles were carefully removed using fine forceps. The longitudinal muscle/myenteric plexus whole-mount preparation included the myenteric plexus adhering to the longitudinal muscle.

### Immunohistochemistry

For studies by light microscopy, free-floating cryostat sections were incubated for 1 h at room temperature with PBST containing 0.1% sodium azide plus 0.5% hydrogen peroxide to inhibit endogenous peroxidase activity (Li et al., 1987), for 12–48 h at 4°C with a primary hpChAT antiserum (diluted 1:10,000) or rabbit antiserum against cChAT (diluted 1:2,000, Kimura et al., 2007), for 1 h at room temperature with biotinylated goat anti-mouse IgG (diluted 1:2,000; BA-2000; Vector Laboratories, CA, USA) or biotinylated goat anti-rabbit IgG (diluted 1:2,000; BA-1000; Vector Laboratories), and lastly for 1 h with avidin-biotin-peroxidase complex (diluted 1:3,000, ABC Elite kit PK-6100; Vector Laboratories). PBST was used to dilute all reagents and wash sections after each step. Color labeling of peroxidase activity was done by reacting the sections for 20 min with 50 mM Tris-HCl buffer (pH 7.6) containing 0.04% 3-3′diaminobenzidine tetrahydrochloride, 0.4% nickel ammonium sulfate, and 0.003% hydrogen peroxide to yield a dark-blue precipitate. Stained sections were mounted on a glass-slide (Platinum Coat, Matsunami Glass, Osaka, Japan), air-dried, washed with tap water, ethanol-dehydrated, cleared in xylene, and cover-slipped with Entellan (Merck, Darmstadt, Germany).

We further tested rabbit antiserum against rpChAT (Tooyama and Kimura, 2000), which has been used for IHC to observe rpChAT-immunoreactive (ir) structures in various animals. When used for IHC in human tissues, however, rpChAT antiserum gave heavy nonspecific diffuse staining. This problem has been solved by using rpChAT antiserum pre-absorbed with an excess amount of human IgG (1 μg/ml of diluted antiserum, Sigma-Aldrich, product number I4506) or human serum (Sigma-Aldrich, S7023, lot 109K8711, 10 μl/ml of diluted antiserum, S. Kimura, personal communication).

For double immunofluorescence (IF) labeling of rpChAT and hpChAT proteins, free-floating cryostat sections of human colon were incubated for 48 h at 4°C with a mixture of rabbit antiserum against rpChAT (diluted 1:400) which had been pre-absorbed overnight at 4°C with human serum, and mouse antiserum against hpChAT (diluted 1:2,000). After washing with PBST, sections were incubated for 3 h at room temperature with a secondary antibody mixture of Alexa Fluor 555-conjugated donkey anti-rabbit IgG (H + L) and Alexa Fluor 647-conjugated donkey anti-mouse IgG (H + L; each diluted 1:500, Thermo Fisher Scientific Inc.). Control sections were subjected to the same procedures without primary antibodies. After washing with PBST, sections were mounted on gelatin-coated glass slides, cover-slipped with immune-Mount (Thermo Shandon, Kalamazoo, MI, USA) and examined using a confocal laser-scanning microscope (FV-1000, Olympus, Tokyo, Japan).

For IF studies on whole mount tissues, two pieces of submucosal plexus or longitudinal muscle/myenteric plexus (~1 cm^2^) prepared from each specimen were used. After washing three times at 10-min intervals with PBS, free-floating preparations were incubated for 30 min at room temperature with 10% normal donkey serum (Jackson ImmunoResearch Laboratories) in PBST, for 5 days at 4°C with hpChAT antiserum (diluted 1:2,000), and for 2 h at room temperature with Alexa Fluor 488-labeled donkey anti-mouse IgG (H + L; diluted 1:400). The Fluor-labeled tissues were then mounted on glass slides with anti-fade mounting media (Vector Laboratories). Images were acquired by using a Zeiss LSM 710 confocal microscope. For IF controls, pre-immune mouse serum (diluted 1:10,000) or diluent alone was used.

For absorption test, 1 ml of hpChAT antiserum (diluted 1:40,000) was incubated overnight at 4°C with 100 μg of one of three peptides; SYKALLD (peptide N5-C6), TQSSRK (peptide N10) and CSYKALLDRTQSSRK (antigen), and then processed for IHC as described above. All synthetic peptides were purchased from Takara Bio Incorporation (Otsu, Japan). Digital images were acquired by using an image acquisition system (DP2-SAL; Olympus) equipped with a camera (DP27; Olympus) attached to a microscope (BX50; Olympus). An image editing software program (Paint.net ver. 4.0.5) was used to crop, trim, and adjust only for contrast and brightness.

The reproducibility of immunostaining data was cross-evaluated by more than two authors in the two laboratories.

## Results

### Specificity and Characterization of hpChAT Antiserum

**Figure 2:**
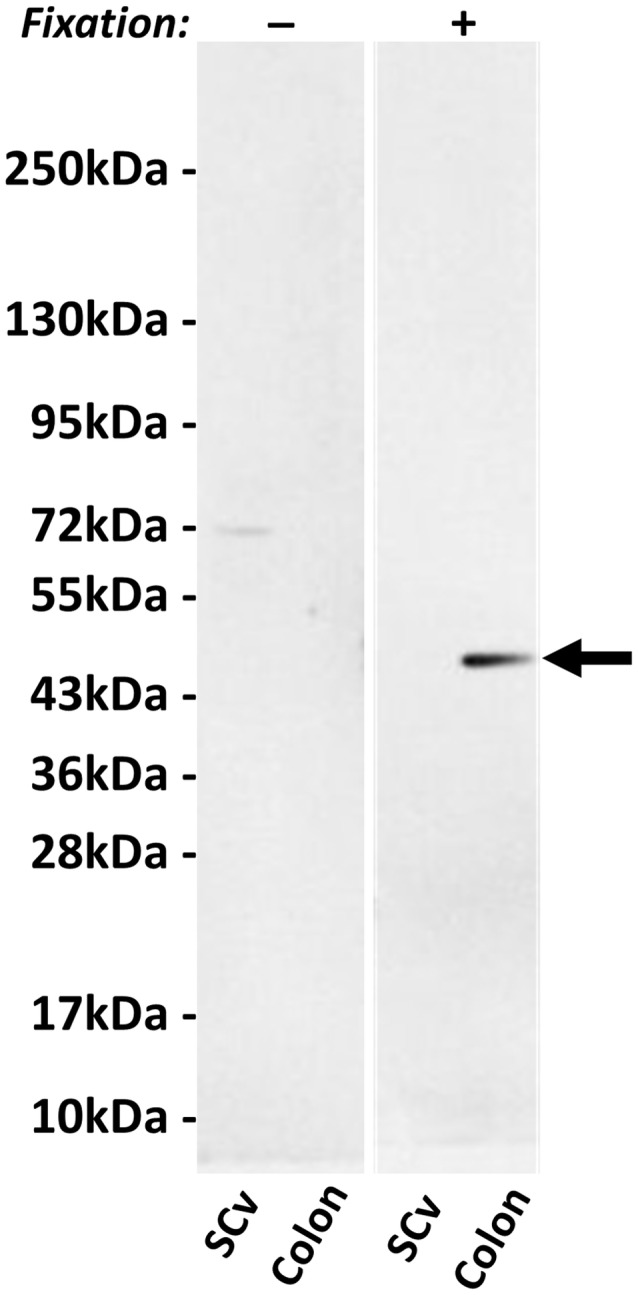
Western blot using hpChAT antiserum on tissue extracts of human ventral spinal cord (SCv) at the cervical level and ascending colon after SDS-PAGE followed by blotting on nitrocellulose membranes with (+) or without (−) paraformaldehyde (PFA) fixation. After fixation, positive hpChAT-immunoreactive (ir) labeling is seen only in a single band of approximately 47 kDa (black arrow) of colon. Molecular weight markers are shown in kDa at the left panel.

Figure 2 shows a Western blot of two human tissue extracts using hpChAT antiserum. In the blotted membrane pre-fixed with PFA, hpChAT-ir staining was seen at a band of approximately 47 kDa in the colon, while no labeling was detectable in the cervical segment of the ventral spinal cord. The size of the band well matched the predicted size of hpChAT. Without fixation, whilst no labeled band was seen in the colon, a faint 72 kDa band was observed in the extract of ventral spinal cord. Thus, we cannot rule out the possibility that hpChAT antiserum may recognize 72 kDa cChAT in the cord under a non-PFA fixed condition. With IHC, however, using human tissues treated with a PFA fixative, the possible detection of cChAT by hpChAT antiserum can be practically denied. Because rpChAT-ir structures have been reported to be densely expressed in the intestine but not ventral spinal cord of rat, the present finding supports the reliability and specificity of the novel antiserum to recognize hpChAT, with no cross-reactivity with any other protein product(s) possibly derived from human *chat* gene.

**Figure 3:**
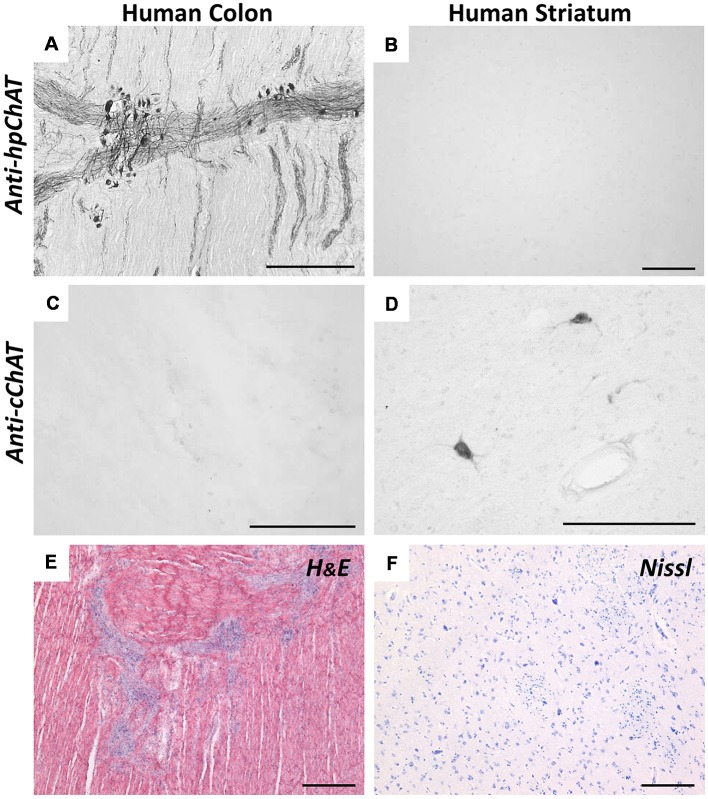
Immunohistochemistry (IHC) using anti-hpChAT **(A,B)** or anti-cChAT **(C,D)** sera in human ascending colon **(A,C)** and striatum **(B,D)**. Note the specific staining with anti-hpChAT serum in colon, and with anti-cChAT in striatum. Hematoxylin-eosin histological staining in the human ascending colon **(E)** and Nissl staining in the human striatum **(F)**. Bars = 200 μm.

Figure 3 illustrates the comparison of staining resulting from IHC with hpChAT and cChAT antiserum in the human ascending colon and brain striatum. In the colon, hpChAT-ir cell bodies and fiber bundles could be seen in nerve plexus and interganglionic strands (Figure 3A), while no staining was observed in the striatum (Figure 3B). In contrast, cChAT-ir staining was undetectable in the colon (Figure 3C), whereas cChAT-ir cells were visualized in the striatum (Figure 3D). These results provided additional support for the specificity of hpChAT antiserum and were consistent with previous studies indicating the preferential distribution of rpChAT in PNS but not CNS cholinergic nerves (Tooyama and Kimura, 2000; Bellier and Kimura, 2007, 2011).

**Figure 4:**
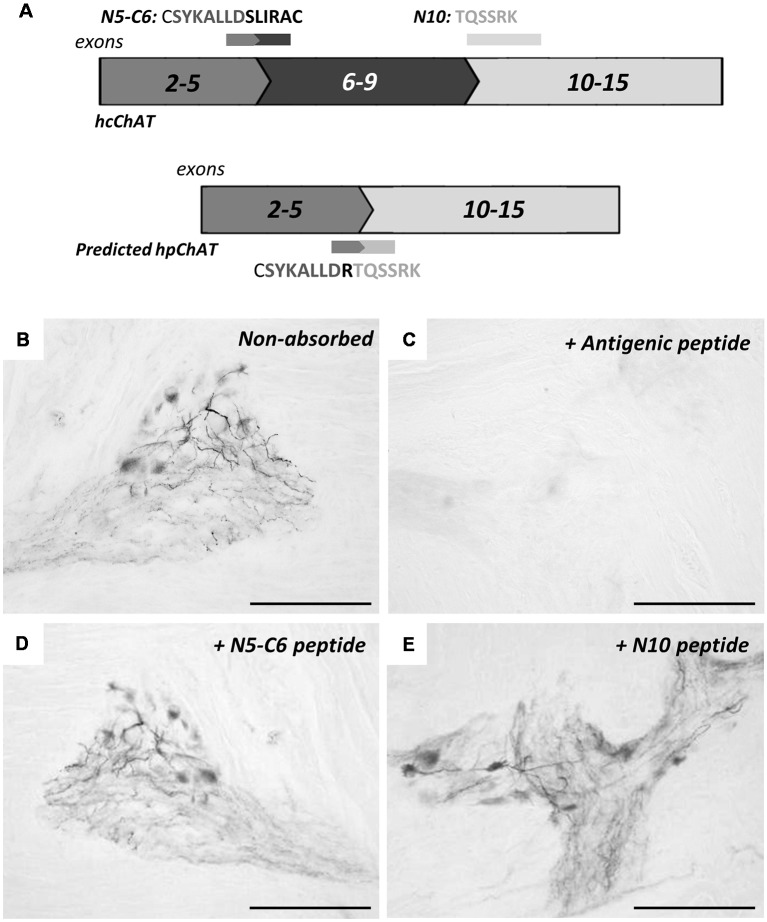
Immunohistochemical absorption test for hpChAT antiserum. Panel **(A)** shows three peptide absorbents chosen; two (C5-N6 and N10) from the full-length spliced exon sequence coding for hcChAT protein (upper panel), and the antigen peptide (see Figure 1) from the splice variant coding for predicted hpChAT protein (lower panel). The exon number is shown in each box. Staining results in the colon using anti-hpChAT antiserum (diluted 1:40,000) alone **(B)**, pre-incubated with the antigen **(C)**, C5-N6 peptide **(D)** and N10 peptide **(E)**. Bars = 150 μm.

Figure 4 shows antibody-absorption tests by IHC in the colon, using three peptides relevant to our interest (Figure 4A). Compared to the non-absorbed control (Figure 4B), hpChAT-ir staining was completely blocked when hpChAT antiserum has been absorbed with the antigenic peptide as expected (Figure 4C). On the other hand, no apparent effects of absorption were detected in terms of the intensity of positive staining in cell bodies and processes with the other two peptides: one contained in cChAT protein (Figure 4D, coded by exons 5–6) and the other contained in both cChAT and pChAT (Figure 4E, coded by 5’ end of exon 10).

**Figure 5:**
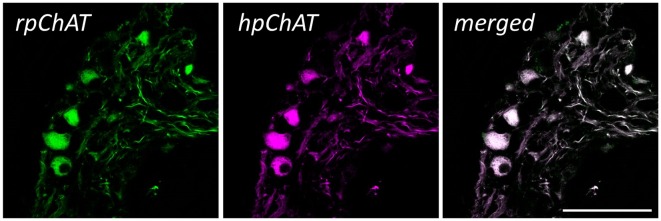
Double immunofluorescence (IF) of rat pChAT (rpChAT) (left panel) and hpChAT-ir (middle panel) structures in submucosal ganglia of human ascending colon. Staining with anti-rpChAT and anti-hpChAT serum. A merged image (right panel) of the two shows a complete overlap of immunoreactivity. Bars = 50 μm.

Figure 5 shows double IF staining in human colon using rpChAT or hpChAT antiserum. As seen in the merged image, both rpChAT-ir and hpChAT-ir molecules always co-existed in a single cell. The result further confirmed the specificity of hpChAT antiserum.

### Distribution of hpChAT-ir Cells and Fibers in Human Colon

**Figure 6:**
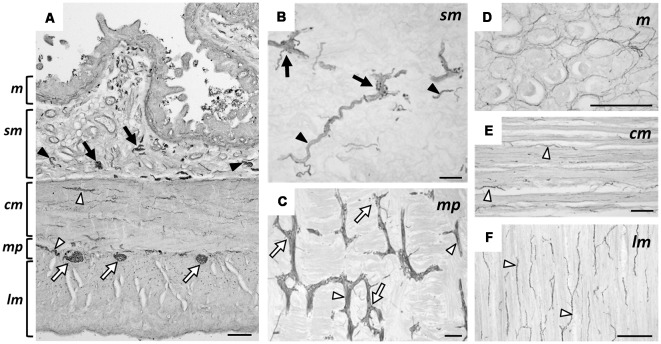
Immunohistochemical staining using hpChAT antiserum in human ascending colon. A transverse section showing the entire layer thickness of colon **(A)**. In **(B–F)**, sections were cut longitudinally parallel to the serosa; positive hpChAT-ir labeling seen in submucosa **(B)**, myenteric plexus **(C)**, mucosa **(D)**, circular muscle layer **(E)**, and longitudinal muscle layer **(F)**. Arrows and arrowheads indicate ganglionated plexus and nerves fibers bundles, respectively, in submucosa (black) and muscularis propria (white). cm: circular muscle, lm: longitudinal muscle, m: mucosa, mp: myenteric plexus, sm: submucosa. Bars = 200 μm.

We present typical hpChAT-ir staining features observed in sections of the human ascending colon (hpChAT staining in human duodenum and ileum are shown in Supplementary Figure S2). Figure 6A shows a transverse section where positive structures were deposited unevenly in certain layers extending throughout the entire wall of the colon. In particular, intense staining could be seen in ganglionic plexus (arrows) and nerve fiber bundles (arrowheads). In longitudinal sections, hpChAT-ir fibers in interganglionic strands and ganglionic cells were seen in the submucosal plexus (Figure 6B) and myenteric plexus (Figure 6C). In the mucosa (Figure 6D), there were bundles of positive fine fibers running irregularly, together with positive single fibers with tiny varicosities in interstitial spaces of the lamina propria. The circular muscle layer contained many stained fine fibers running parallel to muscle fibers (Figure 6E). In the longitudinal muscle layer (Figure 6F), bundles of hpChAT-ir fine fibers were seen, densely near the myenteric plexus and lightly near the serosa, to run also along muscle fibers (data not shown).

**Figure 7:**
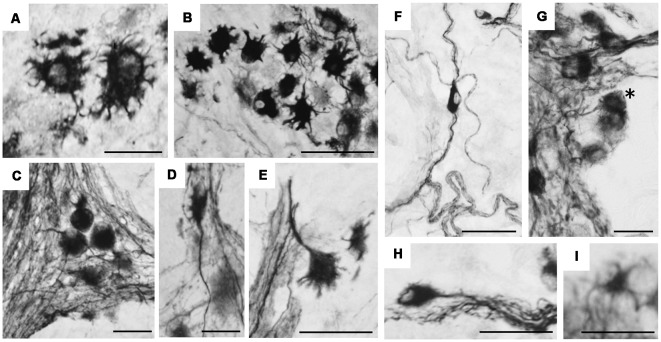
Immunohistochemical staining using hpChAT antiserum in enteric ganglia of human ascending colon. Cell types of positive neurons were identified in myenteric plexus **(A–E)** and submucosal plexus **(F–I)**; Dogiel type I bearing large **(A)** and small **(B)** cell bodies with short multiple dendrites, Dogiel type II having round or oval cell bodies with smooth surfaces and several prominent axonal processes **(C)**, Dogiel type III with protruding axon and short dendrites **(D)**, Dogiel type IV with single axon and short branching dendrites **(E)**, submucosal Dogiel type II **(F)**, Dogiel type IV [asterisk in **(G)**], unipolar **(H)** and stellate cells **(I)**. Bars = 20 μm.

At higher magnification (Figure 7), hpChAT-ir could be seen in the neuronal cytoplasm, while the cellular nucleus appears devoid of staining. Intensely stained hpChAT neuronal cells were found densely in both myenteric and submucosal ganglia. As their dendritic processes were also clearly labeled, we were able to identify their cell types according to the classification described previously (Furness, 2006). Figures 7A–E show Dogiel type I (of either large or small soma), II and III neurons observed in the myenteric plexus, and Figures 7F–I display Dogiel type II and IV neurons as well as unipolar and stellate neurons in the submucosal plexus.

**Figure 8:**
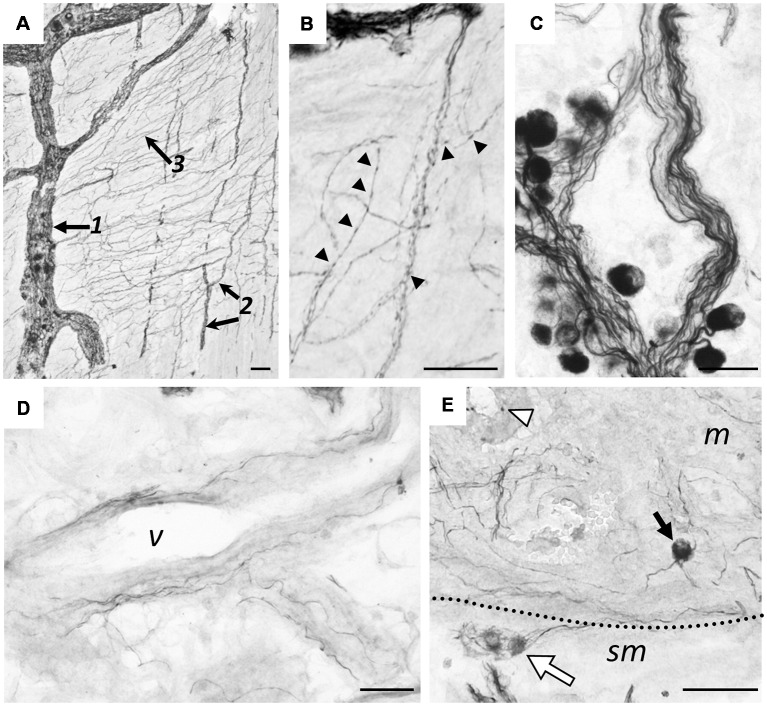
Immunohistochemical staining using hpChAT antiserum in human ascending colon. Positive nerve fibers and cells in the myenteric plexus **(A,B)**, submucosa **(C,D)** and mucosa **(E)**. A section through the myenteric plexus **(A)** shows positive labeling in primary nerve strands (arrow 1), secondary nerve strands (arrows 2) and fine-meshed tertiary nerve network (arrow 3). At a high magnification of the tertiary network **(B)**, fine hpChAT-ir fibers appear to be varicose (arrowheads). In a section through the submucosal plexus, smooth or non-varicose fibers are located in nerve strands **(C)**. Occasionally, smooth positive fibers are seen to run along a blood vessel (v) in the submucosa **(D)**. Panel **(E)** shows two hpChAT-ir submucosal cells (white arrow) and a round positive cell (black arrow) in the mucosa (m) near the submucosa (sm). Bars = 50 μm.

Figure 8 illustrates hpChAT-ir nerve fibers distributed in various regions including myenteric and submucosal plexuses (Figure 8A). We observed in Figure 8B; fine positive fibers running out of myenteric plexuses or their strands mostly bearing tiny swellings. In contrast, axon-like fibers originating from positive cells in the submucosal ganglia were often thick and smooth (Figure 8C). Although most blood vessels in the colon seemed to receive no or few hpChAT-ir fibers, blood vessels in the mucosa seemed to have a few positive thin fibers running irregularly along their vessel wall (Figure 8D). In the mucosa, there were many stained varicose fibers in interstitial spaces of the lamina propria (Figure 8E). The mucosa also contained hpChAT-IR cell bodies, shaped round and scattered often alone (Figure 8E).

**Figure 9:**
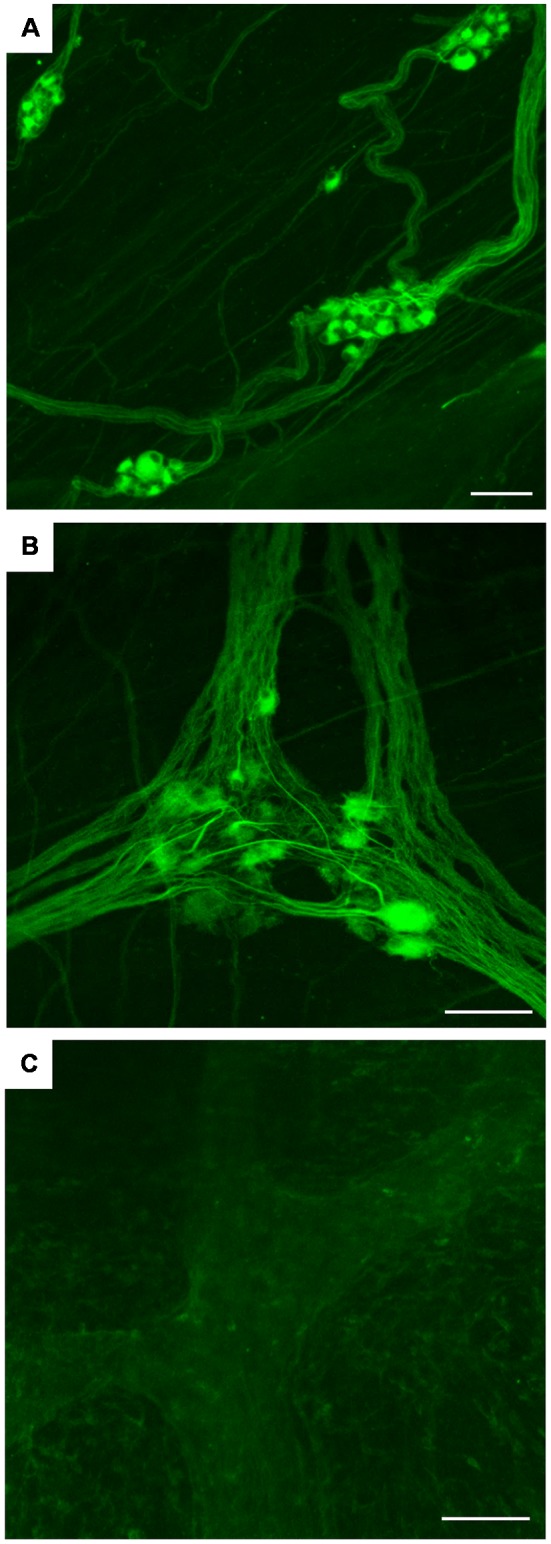
Whole mount preparations of human sigmoid colon. IF using hpChAT antiserum **(A,B)** and vehicle alone **(C)**; inner submucosal plexus **(A)** and longitudinal muscle/myenteric plexus **(B,C)**. The results, being consistent with those in thin cryostat sections (Figures 5–8), indicate that the method using thick tissues will help to improve our understanding of three-dimensional organizations of structures stained with hpChAT antiserum. No specific staining is seen in the control processed without hpChAT antiserum **(C)**. Bars = 100 μm.

By whole-mount IF on human sigmoid colon, hpChAT-ir neurons and fibers were seen in the submucosal plexus (Figure 9A) and longitudinal muscle/myenteric plexus (Figure 9B). The border of each positive cell was distinct and the positive fluorescence, varying in intensity, was confined in the cytoplasm and processes. Both Dogiel type I and type II cells were stained for hpChAT antiserum (Figure 9B). No positive staining was detectable in the control tissue incubated without hpChAT antiserum (Figure 9C).

## Discussion

### Design of Antigenic Peptide for the Production of hpChAT Antiserum

The aim of the present study was to raise a specific antiserum against hpChAT to visualize probable cholinergic neurons in the human PNS. The first challenge we have faced relates to the lack of available information on hpChAT protein sequence. All our attempts to isolate pChAT mRNA from human PNS tissues were unsuccessful (data not shown). The reasons for this failure remain elusive. One possibility is that human samples we analyzed were all collected several hours postmortem. While long-term stability of RNA has been shown in postmortem human brains up to 48 h (Johnson et al., 1986), some RNA species, especially mRNA, are reportedly prone to rapid postmortem degradation (Sidova et al., 2015). Since rpChAT mRNA has been shown to be difficult to isolate even from freshly obtained rat tissues (Matsuo et al., 2005), hpChAT mRNA collected from postmortem human tissues might be more difficult to obtain in high quality.

Then, we have postulated that pChAT, an exon-skipping smaller splice variant of cChAT, is evolutionary conserved among different species, including rats and humans. Evidence to support this hypothesis comes from two approaches. First, our genetic analysis of *chat* genes held in databases shows strong conservation between the rat and human in the splicing pattern as well as the coding region (Figure 1). Second, there is convergent evidence, through studies using rpChAT-IHC supporting the existence of pChAT in various mammals. These include not only the rat but also mouse, pig, guinea-pig, monkey (Brehmer et al., 2004; Chiocchetti et al., 2004; Yuan et al., 2005; Wang et al., 2012; Koga et al., 2013) and, as presented here, human. It is also noteworthy, that pChAT appears to occur also in an invertebrate species, the octopus, as found by rpChAT-IHC (Sakaue et al., 2014; Bellier et al., 2017).

In the present study, we have used a predicted protein sequence of hpChAT to design antigenic peptides to raise specific hpChAT antisera, not cross-reacting with human cChAT. This seems theoretically difficult, as every translated peptide coded by each exon of hpChAT exists in human cChAT. An exception is arginine in hpChAT, which is formed at the splice junction of exon 5 and exon 10. Instead, an amino acid serine coded by alternative spliced exons 5–6, and a valine by exon 9–10 in hcChAT are deleted in hpChAT. A possible solution to overcome this difficulty was identified in the previous study for the production of rpChAT antiserum (Tooyama and Kimura, 2000). They employed a peptide antigen consisting of 41 amino acids corresponding to the mRNA sequence across the splice junction of exons 5 and 10 in rpChAT. Thus the antigen, comprising of a combined peptide linked by arginine, could be a unique target for immune cells to distinguish rpChAT from rat cChAT. In the present study, we adopted a similar strategy to design antigenic peptides by sharply focusing on the amino acid alignment immediately around the splice junction of exons 5 and 10 in our predicted hpChAT. It is interesting to note that the entire peptide (18 amino acids, data not shown) coded by exon 5 of human is almost the same as that of rats, while a sequence highly specific to primates is derived from the beginning (5’ end) of exon 10, namely the eight amino acids (TQSSRKLI) next to the junction. As the pentadecapeptide antigen used here contains seven amino acids (SYKALLD) from exon 5, and six amino acids (TQSSRK) from exon 10, it seems that the latter primate-specific hexapeptide could represent an epitope in hpChAT. However, this assumption is clearly denied in our absorption test (Figure 3); no, if any, change in staining result was seen by IHC using hpChAT antiserum which had been pre-incubated with either SYKALLD (peptide N5-C6) or TQSSRK (peptide N10). In contrast, the absorption test with the antigen consisting mainly of these two peptides resulted in complete elimination of positive staining. It is therefore very likely that hpChAT antiserum recognizes the three-dimensional conformation of 14-amino acids, which might be incorporated in the protein folding of hpChAT. We further suggest that in both human and rat, pChAT differs significantly from cChAT in protein folding. In such a case, an epitope, which is exposed and accessible to antibodies in pChAT, may possibly be buried and inaccessible to them in cChAT. Such differences in protein structure may explain the specificity of the antiserum to hpChAT, having no cross-reactivity with human cChAT.

### Specificity and Characterization of hpChAT Antiserum Studied by Western Blot and IHC

First, to assess our Western blot analysis, we describe information on the isoforms of human cChAT protein. From the human *chat* gene, five transcripts for cChAT (N1, N2, R, S and M) are produced by alternative splicing (Misawa et al., 1997; Ohno et al., 2001). All these variants possess start codons for translation of 70 kDa cChAT protein (isoform 1). The M variant has an additional start codon for 83 kDa cChAT protein (isoform 2) and the S variant has another codon for 74 kDa cChAT protein (isoform 3). All isoforms of cChAT protein contain an identical amino acid sequence coded by exons 3–15, and so the difference in size among the three isoforms depends on differential usage of upstream exons. Because the deduced molecular weights of the isoforms 2 and 3 are larger than the size of ChAT protein purified from human brain (66 kDa, Peng et al., 1980) and placenta (68 kDa, Bruce et al., 1985), the 70 kDa cChAT protein (isoform 1) appears identical with the purified cChAT proteins and is likely the primary product of the human *chat* gene. The isoform 1, also termed isoform CRA_a, consists of 630 amino acids translated from spliced exons 2–15, has been deposited in databases (NP_001136401, D3DX95_HUMAN). Instead, the larger ChAT isoforms have been suggested to be enzyme precursors for processing or to carry N-terminal extra-peptides for subcellular localization (Oda et al., 1992; Misawa et al., 1997). Supporting this latter assumption, the 82 kDa cChAT has been found in nuclei of neuronal cells in human CNS, but not in their cytoplasm or cell membranes as commonly labeled by IHC using many other cChAT antibodies (Gill et al., 2007; Albers et al., 2014). It is, therefore, reasonable to assume that, in human colon, the predicted size of hpChAT protein is 47 kDa (420 amino acids), as a short exon-skipping splice variant of 70 kDa cChAT protein. This postulation is in good agreement with our Western blot where only a single band of approximately 47 kDa is clearly labeled with hpChAT antiserum. Taken together, the novel antiserum recognizes 47 kDa hpChAT, but not other species of hpChAT, if present, or human cChAT, all significantly larger than 47 kDa.

Second, our results by IHC indicate that, at the cellular level, reaction products positive for hpChAT antiserum occur in the cytoplasm of neuronal cell bodies, dendritic processes and axons, but not in the cellular nuclei. At the tissue or organ level, numerous hpChAT-positive neuronal structures are detected in human colon, while no positive staining for hpChAT is seen in the brain striatum and ventral spinal cord, both CNS regions known to contain dense cChAT expression (Woolf, 1991). These staining features appear essentially identical with those reported in various animals by IHC using rpChAT (Tooyama and Kimura, 2000; Bellier and Kimura, 2007, 2011). This notion is further confirmed by the double IF staining using hpChAT antiserum and rpChAT antiserum in human colon, demonstrating that hpChAT and rpChAT proteins always coexist in a single neuronal cell. Importantly, the use of hpChAT-IHC, moreover, permits us to observe detailed morphology of positive cells in human colon. We are able to identify cell types, such as Dogiel type cells in both myenteric and submucosal plexuses. All these hpChAT-positive cells have been shown to be cholinergic in guinea-pigs in functional and pharmacological studies (Furness, 2006; see p32–33).

Third, as pointed earlier, our successful absorption test clearly indicates the specificity of the novel antiserum for detecting hpChAT, which is predicted to contain most of the antigen peptide sequence used here. The most important advantage of hpChAT antiserum compared with rpChAT antiserum comes from the antigenic peptides used. While rpChAT antiserum was raised against a 41-amino acid peptide of rat, hpChAT was against a shorter 14-amino acid human peptide containing a primate-specific 6-amino acid sequence. Therefore, hpChAT antiserum could be very useful for phylogenetic studies of pChAT molecules in species closely related to primates including human. In contrast, as described above, rpChAT antiserum has been shown to crossreact with pChAT-like molecules in a relatively wide variety of animal species, probably due to various antibodies against different epitopes contained in the longer peptide sequence.

Taken together, Western blot, IHC and absorption test findings provide a rational basis to validate the specificity of hpChAT antiserum and its use to visualize probable cholinergic innervation in the human colon, that are otherwise hardly detectable by IHC using available cChAT antibodies. However, the specificity of hpChAT antiserum remains to be further examined by IHC in other non-colonic human tissues, particularly parasympathetic cholinergic ganglia.

### Cholinergic Structures in Human Colon Revealed by hpChAT-IHC

Compared to previous reports by IHC with cChAT antibodies in human intestines (Porter et al., 1996, 2002; Ratcliffe et al., 1998; Neunlist et al., 2003; Beck et al., 2009), the present hpChAT-IHC presents excellent resolution images of stained cells and fibers with high signal-noise ratios. In accordance with studies by cChAT-IHC in human intestines (Porter et al., 1996; Furness, 2006), almost all cells labeled by hpChAT appeared to be neurons. In addition, we are able to observe many hpChAT-ir varicose nerve fibers in both circular and longitudinal muscle layers, supporting the role of intramural cholinergic neurons in ACh-induced contractions of the colon (Fishlock and Parks, 1963). The present method further permits detection of hpChAT-ir nerve fibers in the ascending colon mucosa that was not achieved before. Such mucosal cholinergic nerves have been expected to occur and play a role in secretion and vasodilatation (Porter et al., 1996; Ratcliffe et al., 1998). An additional finding is that hpChAT-ir neuronal cells exist in the mucosa of the ascending colon. Mucosal neurons have been reported to exist in human small and large intestines (Lassmann, 1975; Fang et al., 1993) and to contain another cholinergic marker, acetylcholinesterase as well as NADHP-diaphorase, peripherin, and calretinin (Fang et al., 1993; Kramer et al., 2011). The role of mucosal neurons in sensory function has been proposed by an electron microscopic study (Newson et al., 1979). This proposal appears applicable to the above hpChAT-ir cells, since other studies have reported the occurrence of rpChAT-immunoreactivity in sensory neurons (Yasuhara et al., 2004, 2007a, 2008; Bellier and Kimura, 2007; Hanada et al., 2013) including enteric primary afferent neurons (Chiocchetti et al., 2004).

### Possible Applications of hpChAT Antiserum

Previous studies by IHC using rpChAT antiserum have revealed the distribution of cholinergic cells or fibers in various tissues of the PNS, including the pterygopalatine, intra-laryngeal, ciliary, sympathetic, parasympathetic, intramural enteric and intrinsic cardiac ganglia and iris (Tooyama and Kimura, 2000; Yasuhara et al., 2004, 2007b; Okano et al., 2006; Yuan et al., 2007; Hanada et al., 2013) in the rat, and enteric neurons in the pig (Brehmer et al., 2004). In addition, the localization of rpChAT-ir products has been shown in sensory neurons of the trigeminal and dorsal root ganglia, primary sensory nerves of skin, and sensory afferents to blood vessels (Tooyama and Kimura, 2000; Yasuhara et al., 2004, 2007a, 2008; Okano et al., 2006; Bellier and Kimura, 2007; Hanada et al., 2013). Therefore, the present hpChAT antiserum appears valuable to examine possible cholinergic innervation in the human counterpart tissues. This antiserum may also be applied to similar studies in non-human primates, as anticipated by the fact that human and non-human primates share similar expression patterns of gene, transcript and protein (Enard et al., 2002; Khan et al., 2013; Osada, 2015), and that in mammalian evolution, transcriptome change is slower in nervous tissues than in other organs (Brawand et al., 2011). As a result, the structures of cChAT protein are almost identical among primates including human as seen in databases.

For visualizing human enteric cholinergic cells, IHC using cChAT antibodies have been applied to studies in normal and diseased tissues (Porter et al., 1996, 2002; Ratcliffe et al., 1998; Neunlist et al., 2003; Beck et al., 2009). In addition, rpChAT antiserum has been utilized in human and animal model studies to clarify the cholinergic roles in the PNS (Tangsucharit et al., 2012; Wang et al., 2012; Duboc et al., 2016; Giancola et al., 2017; Rice et al., 2017; Uranga et al., 2017). For future disease-oriented studies, the hpChAT antiserum may become a novel tool to analyze morphological changes in peripheral cholinergic structures underlying pathological conditions in humans.

## Author Contributions

J-PB, IT and HK contributed to the conception and methodology. J-PB and HK raised and characterized antisera. J-PB, P-QY, KM and HK contributed to the acquisition, analysis and validation of the data. J-PB wrote the original draft. P-QY, YT and HK reviewed and edited it critically for important intellectual content. All authors listed provide approval for publication.

## Conflict of Interest Statement

HK, J-PB and IT have a patent pending (Japanese 04412018) Antibody for detecting human peripheral cholinergic nerve (2018). The remaining authors declare that the research was conducted in the absence of any commercial or financial relationships that could be construed as a potential conflict of interest.
