# Supplementary material for: A Novel Antiserum Against a Predicted Human Peripheral Choline Acetyltransferase (hpChAT) for Labeling Neuronal Structures in Human Colon
Source: Front Neuroanat. 2019 Apr 16;13:37. doi: 10.3389/fnana.2019.00037 (PMC6476985; doi:10.3389/fnana.2019.00037)
Supplement: Supplementary file 1 [file Data_Sheet_1.docx]

Supplementary Material

# Supplementary Figures


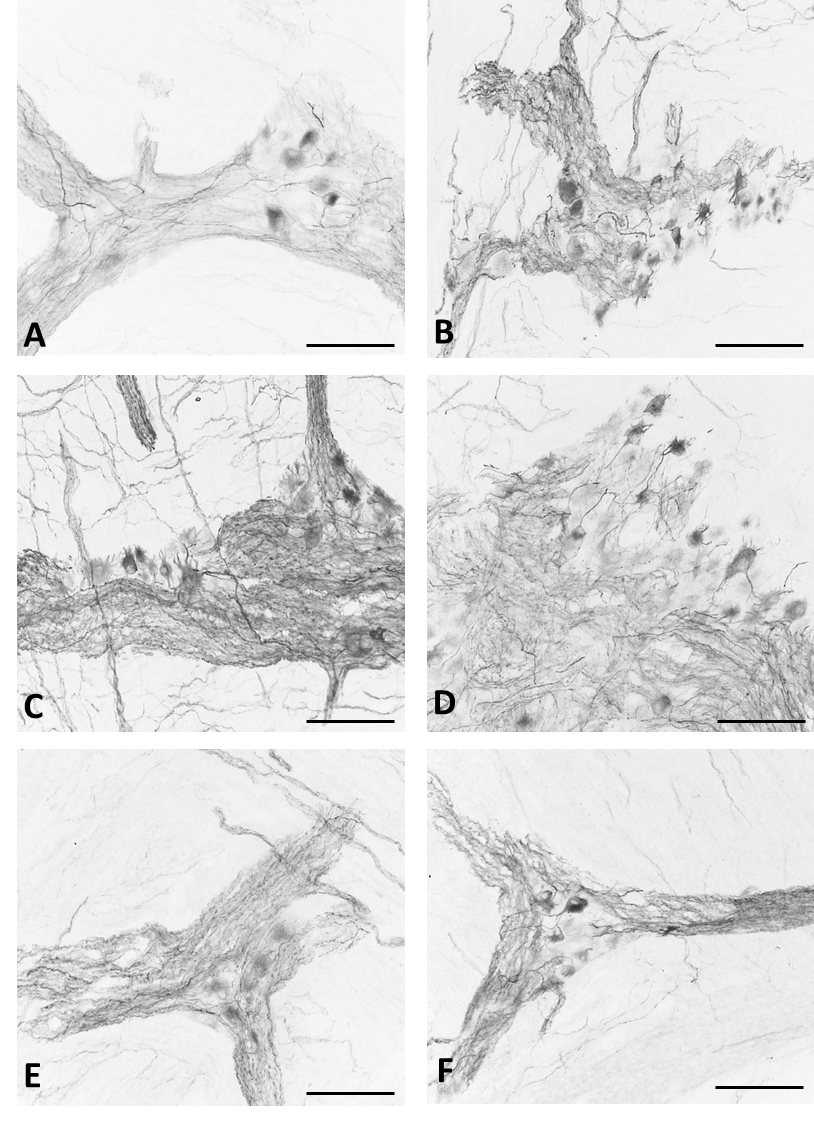


**Supplementary Figure 1.** (A-F) IHC on human ascending colon section using hpChAT antisera from individually immunized mice (dilution 1:20,000). IHC with H3 antiserum used in the present study is illustrated in C. All scale 100 μm.

**
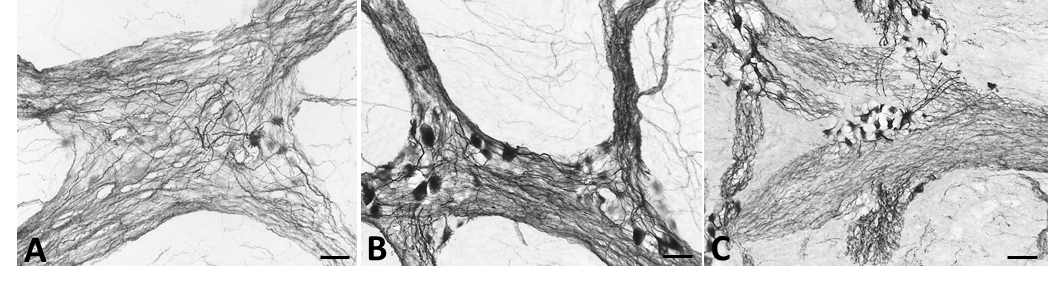
**

**Supplementary Figure 2**. Immunostaining using hpChAT antiserum in the human (A) duodenum, (B) ileum, and (C) ascending colon. All scales = 50 μm.
